# Novel model for end-neuroma formation in the amputated rabbit forelimb

**DOI:** 10.1186/1749-7221-5-6

**Published:** 2010-03-18

**Authors:** Peter S Kim, Jason Ko, Kristina K O'Shaughnessy, Todd A Kuiken, Gregory A Dumanian

**Affiliations:** 1 Department of Surgery, Division of Plastic and Reconstructive Surgery, Northwestern University, Feinberg School of Medicine, Chicago, IL, USA; 2 Neural Engineering Center for Artificial Limbs (NECAL), Rehabilitation Institute of Chicago, Chicago, IL, USA

## Abstract

**Background:**

The forelimb amputee poses many reconstructive challenges in the clinical setting, and there is a paucity of established surgical models for study. To further elucidate the pathogenic process in amputation neuroma formation, we created a reproducible, well-tolerated rabbit forelimb amputation model.

**Methods:**

Upon approval from the Institutional Animal Care and Use Committee, 5 New Zealand White rabbits underwent left forelimb amputation. During this initial surgery, the median, radial and ulnar nerves were transected 1.6-2.5 (mean 2.0) cm distal to the brachial plexus, transposed onto the anterior chest wall and preserved at length. Six weeks subsequent to the amputation, the distal 5 mm of each neuroma was excised, and the remaining stump underwent histomorphometric analysis.

**Results:**

The nerve cross sectional areas increased by factors of 1.99, 3.17, and 2.59 in the median (p = 0.077), radial (p < 0.0001) and the ulnar (p = 0.0026) nerves, respectively. At the axonal level, the number and cross-sectional area of myelinated fibers demonstrated an inverse relationship whereby the number of myelinated fibers in the median, radial and ulnar nerves increased by factors of 5.13 (p = 0.0043), 5.25 (p = 0.0056) and 5.59 (p = 0.0027), and the cross-sectional areas of these myelinated fibers decreased by factors of 4.62 (p < 0.001), 3.51 (p < 0.01), and 4.29 (p = 0.0259), respectively.

**Conclusion:**

Given that the surgical model appears well-tolerated by the rabbits and that patterns of morphologic change are consistent and reproducible, we are encouraged to further investigate the utility of this model in the pathogenesis of neuroma formation.

## Introduction

In the modern era of military combat, there is an increasing incidence of extremity amputations [1], and though advances in body armor and trauma resuscitation have allowed soldiers to survive previously mortal wounds, the cost of survival is often a mangled or amputated extremity. Neuroma formation from amputated nerve stumps can create a challenging clinical scenario since successful treatment of painful neuromas is often elusive [2-4], due in part to the activity of the regenerating nerve fibers at the amputation site.

Two key histologic characteristics of the end-neuroma are the sprouting of nerve fibers in the regenerating growth cone and a preponderance of dense collagen and fibroblastic stroma [5-7]; however, quantifying the histologic characteristics of end-neuromas is difficult to perform in a reproducible manner. Currently in the literature, there is no forelimb amputation animal model that results in an end-neuroma; however, such a model is necessary to further characterize key histologic parameters of the end-neuroma and to guide further medical and surgical modalities in the treatment of painful neuromas. In this study, we describe a novel end-neuroma model in the amputated rabbit forelimb which has not been previously described in the literature. The advantages of this model include the following: 1) amputation of the forelimb and transection of the median, radial and ulnar nerves creates an environment that approximates the milieu seen in the human proximal upper extremity amputation; 2) a surgical technique that minimizes animal morbidity; and 3) the creation of an end-neuroma with quantifiable and reproducible histomorphometric parameters.

## Materials and methods

The surgical and animal care protocol was created in close collaboration with the Center for Comparative Medicine to ensure full compliance with Institutional Animal Care and Use Committee (IACUC). In order to maximize the number of nerve specimens per animal, and thereby minimize the number of actual animals used in the study, three end-neuromas were created in the forelimb. The animal husbandry staff at our institution felt that functional denervation of the rabbit forelimb with preservation of the extremity would cause undue morbidity. Therefore, in an effort to also minimize the potential for autotomy and its ensuing would complications, a true, as opposed to a physiologic, amputation was performed. Limb amputation in the New Zealand White rabbit has been has been described [8-10], but not been utilized in the investigation of end-neuroma formation. Accordingly, five 6-month old female New Zealand White rabbits (approximate weight 2.5-3.5 kg) were acquired (Covance Inc., Princeton, NJ) and housed one animal per cage in a barrier facility. The animals were housed a minimum of one week prior to initiation of the experiment to allow proper acclimation. All animals were handled in accordance with the guidelines established by the Northwestern University IACUC.

### Operative Technique

Prior to the surgical procedure, the animal was given pre-operative injections of enrofloxacin 5 mg/kg subcutaneously (SQ) (Baytril^®^, Bayer HealthCare LLC, Animal Health Division, Shawnee Mission, KS) and buprenorphine 0.05 mg/kg SQ (Buprenex^® ^Injectable, Reckitt Benckiser Pharmaceuticals Inc., Richmond, VA) and then sedated with ketamine 40-45 mg/kg intramuscularly (IM) (Ketaset^®^, Fort Dodge Animal Health, Overland Park, KS) and xylazine 5-7 mg/kg IM (X-ject E, Butler Animal Health Supply, Dublin, OH). The animal's left anterior and posterior thorax, shoulder girdle, and entire left forelimb, were shaved and treated with a depilatory cream. An intravenous catheter was inserted into a lateral ear vein, through which crystalloid intravenous fluids were administered throughout the procedure, and anesthesia induction was achieved with 5% isoflurane (IsoThesia, Butler) in 100% oxygen (O_2_) through mask ventilation. An endotracheal tube was inserted and used to maintain general anesthesia using 2-4% isoflurane in 100% O_2 _throughout the procedure. Core body temperature was maintained using a circulating-water heating pad.

**Figure 1:**
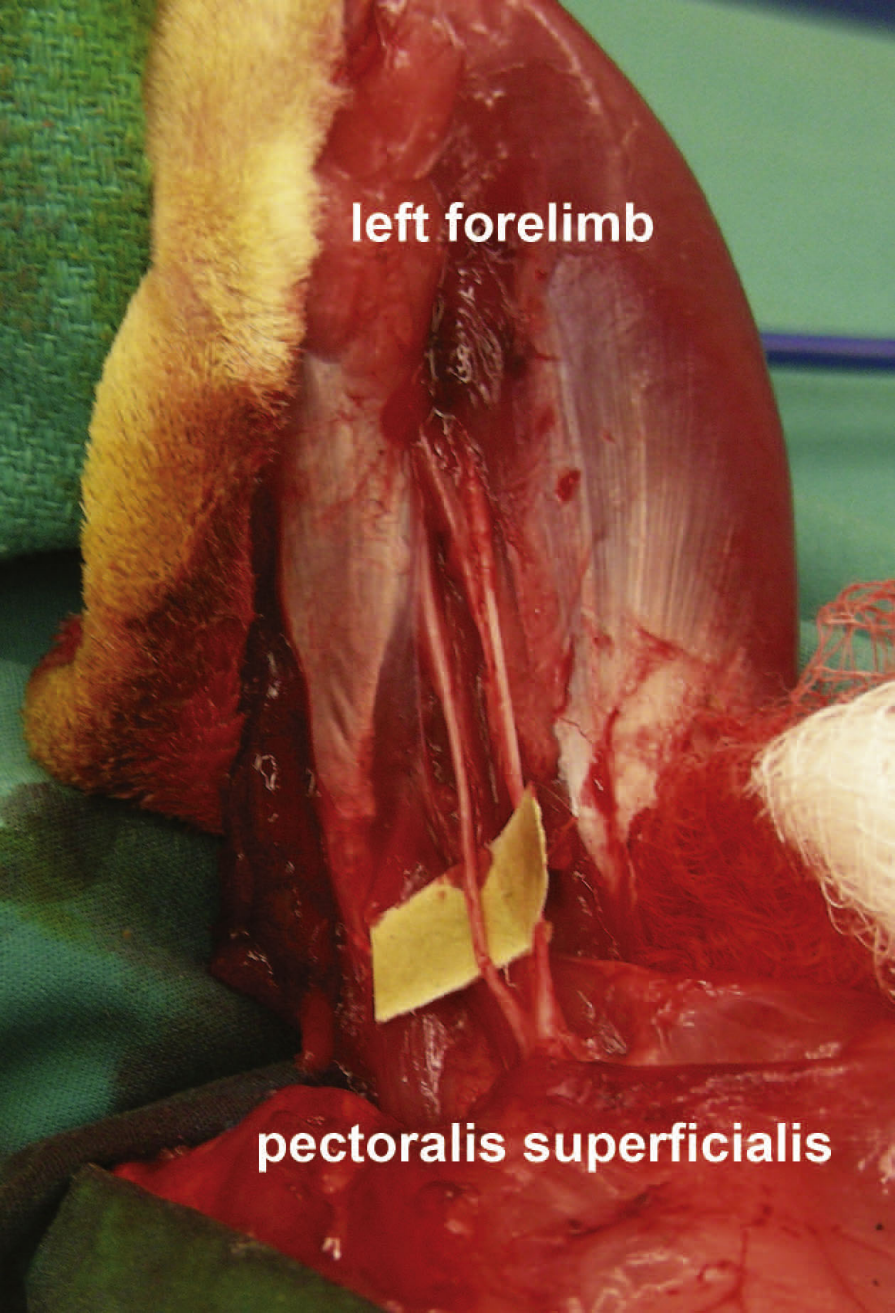
**An elliptical incision was made around the left forelimb of the rabbit**. The pectoralis superficialis is disinserted and with the limb abducted, the nerves exiting the brachial plexus are visualized (the median nerve is highlighted using a background).

**Figure 2:**
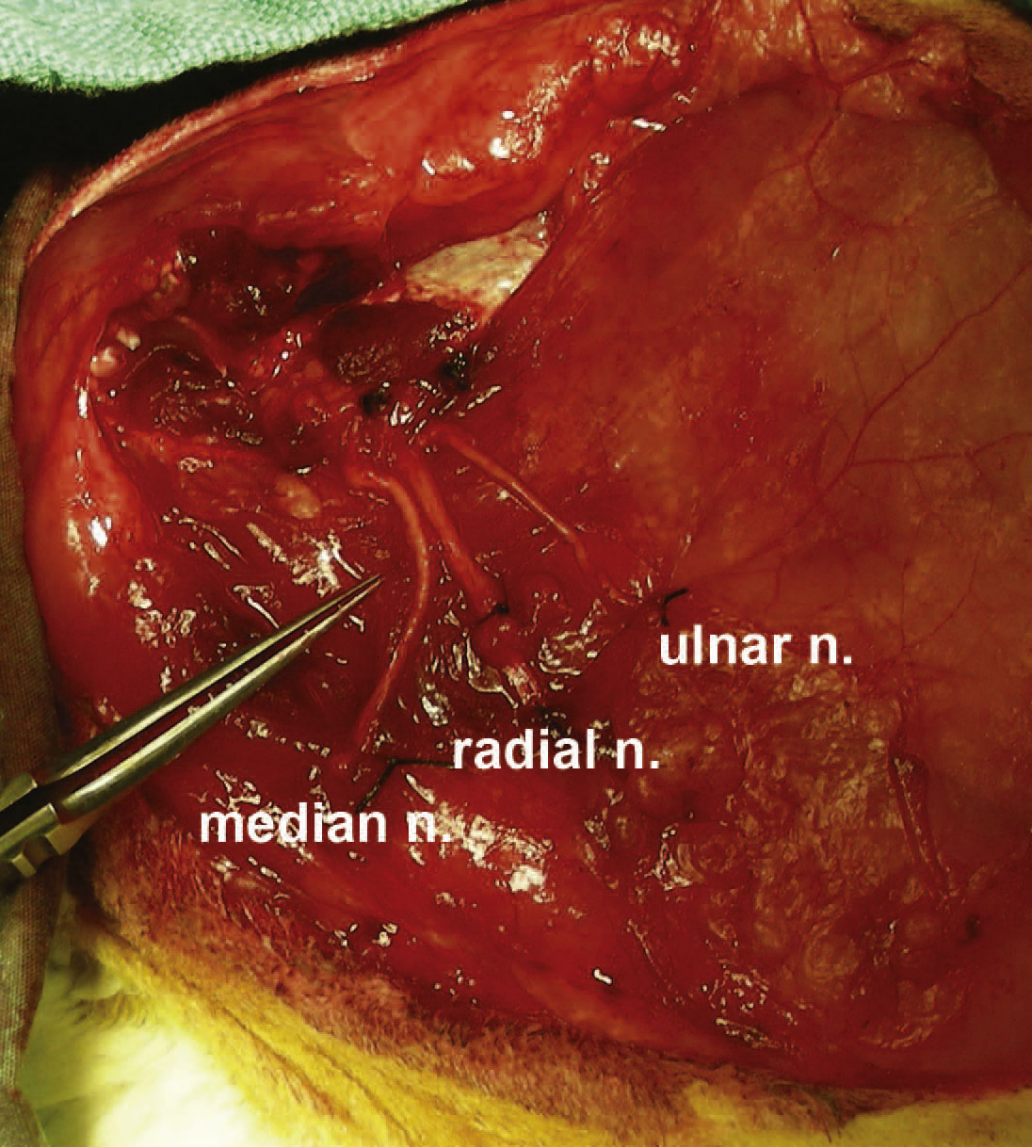
**The median, radial and ulnar nerves are carefully identified**. When possible, the epineurium sutured to the pectoralis fascia on the anterior chest wall with 7-0 Prolene sutures.

An elliptical skin incision was made around the left forelimb girdle, encompassing the axillary fold medially in anticipation of primary skin closure subsequent to the amputation (Figure 1). With the forelimb abducted and extended, careful dissection was performed to expose and identify all nervous structures as they exited the brachial plexus, with special attention directed to the median, radial, and ulnar nerves. Care was taken to preserve and preferentially ligate the brachial artery distally. The median, radial, and ulnar nerves were each transected 2 cm distal to where they branched off of the brachial plexus--the distal nerve segments were harvested for histomorphometric analysis, whereas the proximal nerve stumps were subsequently splayed out and when possible the epineurium sutured to the anterolateral aspect of the pectoralis superficialis transversus muscle fascia using 7-0 polypropylene suture (Prolene suture, Ethicon Inc., Somerville, NJ) (Figure 2). The cut nerve endings were effectively fixed in position on the fascia and not truly implanted into the muscle. The pectoralis and deltoid muscles were then disinserted from the humerus, and all tendinous insertions and muscle fibers were divided at the level of the proximal humerus. Using a scalpel to disrupt the remaining ligaments at the glenohumeral joint, shoulder disarticulation was performed, and all remaining soft tissue attachments were divided in order to complete the forelimb amputation. The remaining pectoralis and deltoid muscles were sutured together using 4-0 polyglactin (Vicryl suture, Ethicon) in order to cover the glenoid fossa and any remaining bony prominences. After meticulous hemostasis was achieved, the elliptical skin incision was closed in a running buried subcuticular fashion using 4-0 polyglactin suture.

### Post-Operative Care

After completion of the procedure, the rabbit was given meloxicam 0.02 mg/kg SQ (Metacam^®^, Boehringer Ingelheim Vetmedica Inc., St. Joseph, MO) and extubated. After the rabbits were allowed sufficient recovery from anesthesia, Elizabethan collars were placed to protect the surgical site and the animals were returned to their cages. The Elizabethan collars were continued for 2-3 weeks post-operatively, and the post-operative medication regimen included meloxicam once daily for 3 days, buprenorphine 2-3 times daily for 3 days, and enrofloxacin once daily for 5 days, in doses previously outlined. The rabbits were inspected routinely throughout the day for abnormal activity, evidence of pain, and post-operative wound complications. The Center for Comparative Medicine staff was in communication with the research staff at 4-8 hour intervals for the first 5 days after surgery to ensure the animals remained well-resuscitated and appropriate levels of analgesics were administered.

### Tissue Harvest and Preparation

**Figure 3:**
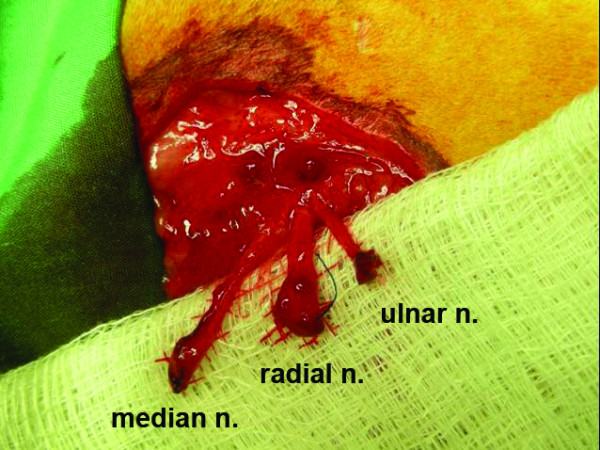
**Six-weeks post-amputation, the surgical site is re-explored**. After careful dissection, the distal stumps of the median, radial and ulnar nerves can be clearly identified.

Six weeks post-amputation, the rabbits were sedated with ketamine 40-45 mg/kg IM and xylazine 5-7 mg/kg IM, and in a fashion similar to the initial surgery, the left forelimb and thorax were shaved and treated with a depilatory cream. Euthanasia was performed with an intracardiac injection of pentobarbital sodium (780 mg/kg) and phenytoin sodium (100 mg/kg) (Euthasol^®^, Virbac AH Inc., Fort Worth, TX). The original surgical incision was re-opened, and the median, radial, and ulnar neuromas and associated nerves were carefully dissected out and brought to length (Figure 3). The distal 5 mm of the nerve and grossly appearing neuroma were excised and discarded. The remaining stump was then sectioned and harvested for histomorphometric analysis.

Harvested nerves were fixed in 4% EM grade glutaraldehyde (Polysciences Inc., Warrington, PA) at 4°C, post-fixed with 2% osmium tetroxide (Polysciences) and serially dehydrated in ethanol. Specimens were embedded in Poly/Bed^® ^812 BDMA (Polysciences) and cut into 1-μm cross-sections with a Leica Ultracut UCT ultramicrotome (Leica Microsystems Ltd., Wetzlar, Germany). Sections were then stained with 1% toluidine blue, and mounted and cover-slipped for imaging.

### Histomorphometric Analysis

A Nikon DS-5M-U1 (Nikon Instruments Inc., Melville, NY) digitizing camera was mounted onto a Nikon Eclipse 50i (Nikon) microscope with a manually controlled stage. Nikon NIS-Elements BR 2.3 imaging software was used to perform nerve histomorphometric analysis of all slides. To prevent sampling and size-related bias introduced by capturing data in small fields and then extrapolating these data to the entire nerve, an image of the nerve was produced using the stitch function of the software (Nikon NIS-Elements). This allowed for a series of high-powered fields to be digitally captured then precisely interlaced. The resulting high magnification composite of the entire nerve was then processed for histomorphometric analysis. Using a semi-automated technique, characterized by dynamic thresholding and manual fiber elimination, [10,11] each nerve was analyzed to determine the nerve cross-sectional area, the myelinated fiber count in each nerve cross-section, and the cross-sectional areas of the myelinated fibers. When non-nerve areas of the field were inadvertently stained by the toluidine blue and osmium tetroxide, and then measured by the software, they were manually stricken from the database by direct inspection and visualization.

On several samples, the nerves were embedded and cut on a bias, creating cross-sections that resembled ovals more than circles. In such circumstances, the slides were discarded and the blocks were recut in order ensure true orthogonal sections of the specimens. Otherwise, all stained myelinated fibers were included in the database for analysis.

In order to prevent grading bias, the observer was blinded to the origin of the prepared slides.

### Statistical Analysis

Pre-amputation controls and post-amputation neuromas from each nerve (median, radial and ulnar) were compared using the two-tailed Student's *t*-test to analyze the following histomorphometric parameters: 1) nerve cross-sectional area; 2) myelinated fiber count; and 3) myelinated fiber cross-sectional area. A *p*-value of 0.05 was considered statistically significant.

## Results

All animals tolerated the amputation well with no post-operative complications and were able to ambulate without difficulty despite the amputation. The animals did not display any behavioral patterns that indicated distress and were able to maintain their body weight throughout the course of the study. No animals were euthanized prior to completion of the study.

**Figure 4:**
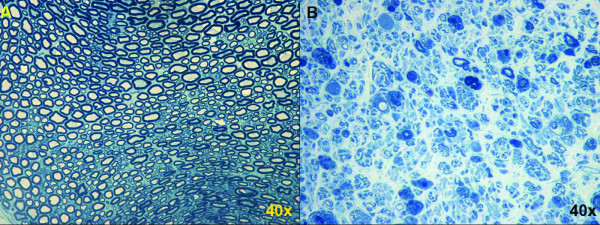
**Side-by-side comparison of the median nerve pre- and post-amputation seen at the same magnification**. Notable changes include a higher proportion of stromal tissue in the neuromas (B) as well as an array of smaller, disorganized myelinated fibers.

Qualitatively speaking, the uninjured nerve and neuromas demonstrate several key characteristics. Figure 4 demonstrates a side-by-side comparison of the median nerve pre- and post-amputation seen at the same magnification. Notable changes include a higher proportion of stromal tissue in the neuromas as well as an array of smaller, disorganized myelinated fibers (Figure 4b). Quantitative analyses corroborate the qualitative observations as seen below.

### Nerve Cross-Sectional Area

**Figure 5:**
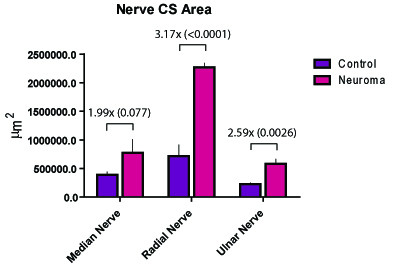
**The mean cross-sectional area of the medial, radial and ulnar nerves measured pre-amputation and at 6 weeks post-amputation**. (P-values provided in parentheses.)

At the time of the harvest, the entire length of the nerve was preserved including the distal neuromatous bulb. During tissue preparation, the grossly appearing distal bulb was excised and discarded. The average cross section area of the distal median, radial and ulnar nerves is demonstrated in Figure 5. The nerve cross sectional areas increased by factors of 1.99, 3.17, and 2.59 in the median (p = 0.077), radial (p < 0.0001) and the ulnar (p = 0.0026) nerves, respectively.

### Myelinated Fiber Count

**Figure 6:**
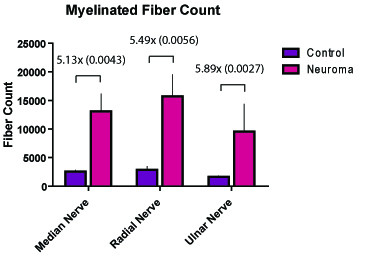
**The number of myelinated fibers quantified in the median, radial and ulnar nerves pre-amputation and at 6 weeks post-amputation**. (P-values provided in parentheses.)

The number of myelinated fibers was quantified at the same level where the nerve cross-sectional area was calculated. Fiber counts demonstrated a statistically significant increase in the number of fibers in the nerves as a result of the amputation (Figure 6). The post-amputation median, radial, and ulnar nerves had 5.13-fold, 5.25-fold, and 5.59-fold the number of fibers when compared with their uninjured controls.

### Myelinated Fiber Cross-Sectional Area

**Figure 7:**
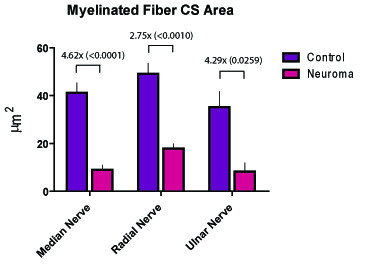
**The average cross-sectional area of myelinated fibers seen in the pre-amputation and post-amputation median, radial and ulnar nerves**. (P-values provided in parentheses.)

At the axonal level, the number and cross-sectional area of myelinated fibers demonstrated an inverse relationship. Although the number of counted fibers increased subsequent to the amputation, the cross-sectional area of these fibers was found to be significantly diminished by factors of 4.62, 3.51, and 4.29 in the median, radial and ulnar nerves, respectively (Figure 7).

## Discussion

Complete physiologic disruption of the nerve, or neurotmesis, leads to a sequence of regenerative and degenerative changes as classically described by Waller [12], during which the proximal nerve stump generates axonal sprouting that occurs both in a collateral and a terminal fashion [13]. Collateral sprouting occurs with the outcropping of nerve fibers proximal to the transection site, typically originating along the nodes of Ranvier [14,15]. In contrast, terminal sprouting is initiated when the transected axons send signals in a retrograde fashion to the cell body, evoking increased cellular metabolism and anterograde transport of neurotrophic peptides. Within 24 hours of injury, a single axon will generate multiple mechanically excitable unmyelinated axon sprouts, constituting a regenerating unit [16]. In addition, a certain cohort of axons perishes due to lack of target-derived neurotrophic support [17-19]. In sum, these collateral and terminal sprouting nerve fibers seek a distal target in order to reestablish the functionality of the nerve.

In the setting of an amputation with end-neuroma formation, a distal target is not established. The axon sprouts then progress to haphazard, randomly oriented myelinated nerves in a rich connective tissue stroma [20]. Therefore, three histologic observations can be made in a mature end-neuroma: 1) The absolute number of myelinated fibers per nerve is increased; 2) the size of each of these myelinated fibers, representing mature nerve sprouts, is diminished compared to the size of healthy axons; and 3) the cross-sectional diameter of the nerve increases as the nerve progresses to a neuroma.

Similar to previous descriptions of neuromas in rabbits [20], 6 weeks was used as a threshold for neuroma formation in this investigation. Six weeks subsequent to forelimb amputation and nerve transection, the surgical site was re-explored, and the distal stumps of the nerves were identified. Each of the nerves was transected 5 mm proximal to the distal tip of the end-neuroma, and the remaining nerve stump was processed for histomorphometric analysis. The results demonstrated that the nerve cross-sectional areas increased by factors of 1.99, 3.17, and 2.59 in the median (p = 0.077), radial (p = 0 < 0.0001) and ulnar (p = 0.0026) nerves, respectively. At the axonal level, the number and cross-sectional area of axons demonstrated an inverse relationship whereby the number of myelinated fibers in the median, radial and ulnar nerves increased by factors of 5.13 (p = 0.0043), 5.25 (p = 0.0056) and 5.59 (p = 0.0027), respectively, and the cross-sectional areas of these myelinated fibers decreased by factors of 4.62 (p < 0.001), 3.51 (p < 0.01), and 4.29 (p = 0.0259), respectively. An additional qualitative observation was that the myelinated fibers in the end-neuroma were amorphous and poorly organized.

The findings of this study are reproducible and comparable to previous descriptions in the literature. The gross neuroma, as measured by increased nerve cross-sectional area, is likely attributable to increased amounts of connective tissue stroma related to inflammation and increased collagen deposition [21]. Since the osmium tetroxide preferentially stains myelinated fibers, the histomorphometric analysis was able to detect an increase in the number of myelinated fibers that were smaller in diameter in a statistically significant fashion. The likely explanation for this observation is that each transected parent axon gives rise to an average of 5 sprouts, as similarly observed by Morris [22]. Due to our use of osmium tetroxide staining for quantification, this study does not account for *un*myelinated fibers. In their electron microscopic analysis of painful human neuromas, Cravioto *et al*. demonstrated that there were significantly increased numbers of unmyelinated fibers relative to myelinated fibers in the distal neuroma segment. Their supposition was that these unmyelinated fibers contributed to the neuropathic pain seen in their study subjects [21]. Of note, the findings in our study study were measured at a static length from the distal end of the neuroma. Further investigation is necessary to elucidate how the histologic topography of the neuroma changes distally to proximally, in a retrograde fashion, particularly when compared to the gross appearance of the nerve proximally.

There are a significant number of animal neuroma models published in the literature. One unique characteristic of the proposed model is that it involves a true amputation as opposed to a physiologic amputation seen in many neuroma-in-continuity models [13,14,21-25]. Removal of the distal nerve segments and the limb recreates the milieu of a human upper extremity amputation, in which the proximal nerve stumps have no distal nerve or motor unit to serve as a target, while avoiding the possibility of autotomy of the remaining denervated limb that occurs after nerve transection in certain animal species. In addition, this model creates an end-neuroma as opposed to a neuroma-in-continuity of the forelimb [26,27] that is reproducible and compares favorably with historic controls.

The goal of this model was to create a reproducible end-neuroma subsequent to forelimb amputation. The histomorphometric analysis of this model was performed in a quantitative fashion. As we implement this model in the investigation of treatment of end neuromas, we intend to use additional qualitative methods to characterize the connective tissue stroma of the neuroma, and the molecular composition of the axon itself. Such methods will include collagen-specific staining, as well as various immunohistochemical stains for axonal markers such as anti-S100 and anti-GFAP. We intend to use the described quantitative model to directly compare commonly used methods of neuroma treatment in the future.

In addition to pathologic changes in neuroma formation, pain is an important clinical manifestation of peripheral nerve injury. In the clinical setting, amelioration of neuropathic pain is often the single metric used to measure treatment success. Although this is a difficult variable to measure in animal models, a recent description of the tibial neuroma transposition (TNT) model of neuroma pain demonstrates that this can be performed in a quantifiable manner [28]. Compared to previously described models, this neuroma pain model measures and differentiates what the authors describe as "neuroma tenderness" from hyperalgesia in the distribution of the injured nerve. We know from clinical practice that amputated limbs may result in paresthesias and localizable tenderness in proximity of severed nerve endings (independent of phantom limb sensation). However, with complete limb amputation in the rabbit, testing for neuroma tenderness is limited to changes in behavior as opposed to measurement of limb withdrawal force as described by Dorsi, et al. Though behavioral methodologies can lend insight into pain, they can be problematic and were thought to be outside the scope of this particular investigation.

As our laboratory continues to investigate the biology of traumatic amputation of the upper extremity, we hoped to develop an amputation model in a large animal species that would enable us to focus on how large peripheral end-neuromas can be modulated surgically and pharmacologically in order to 1) alter neuroma formation; and 2) optimize the transected nerve for applications in neural-machine interfacing. With our proposed model, these goals were achieved while minimizing morbidity to the animal. Additionally, phantom limb and neuropathic pain are phenomena that have complex neurophysiologic and neuropsychologic underpinnings. Further investigation using this model would also help ascertain whether surgical and pharmacologic manipulation of the end-neuroma can effectively alter the experience of this pain.

## Conclusions

We describe a novel rabbit forelimb amputation model that reliably and predictably produces end-neuromas. In addition, the histomorphometric data gathered from this model are comparable to similar reports in the literature. For these reasons, this amputation model will serve as an important platform for future investigation into the behavior and response of traumatic neuromas in the upper extremity.

## Competing interests

The authors declare that they have no competing interests.

## Authors' contributions

PK participated in design and execution of the model and drafting of the manuscript. JK carried out nerve imbedding and histomorphometric analysis and preparation of the manuscript, KO engineered the imbedding and histomorphometric techniques specific for the needs of this model, TK and GD participated in the design and coordination of the model. All authors read and approved the final manuscript.
